# The molecular pathogenesis of SOX2 in prostate cancer

**DOI:** 10.1007/s12672-025-01972-y

**Published:** 2025-02-20

**Authors:** Shixue Liu, Honglian Yu, Zhankui Zhao

**Affiliations:** 1 Jining Medical University, Jining, 272067 Shandong China; 2 Department of Biochemistry, Jining Medical University, 133 Hehua Road, Jining, 272067 Shandong China; 3 Department of Urology, Affiliated Hospital of Jining Medical University, Jining Medical University, 89 Guhuai Road, Jining, 272029 Shandong China; 4 Department of Urology, Seventh People’S Hospital of Shanghai University of TCM, Shanghai, 200137 Shanghai China

**Keywords:** SOX2, Prostate cancer, Pathogenic mechanism

## Abstract

SOX2 is one of the members of the SOX transcription factor family, which is believed to be an important transcription factor that plays a role in embryonic development, maintenance of stem cells, cancer progression, and resistance to cancer treatment. There is increasing evidence suggesting that SOX2 is crucial for the initiation, progression, invasion, metastasis, and treatment resistance of prostate cancer, therefore understanding the mechanism of SOX2 in prostate cancer can provide better targets for the treatment of prostate cancer. This article reviews the structural domains, normal physiological functions, and role in prostate cancer progression of SOX2, providing potential targets for prostate cancer treatment.

## Introduction

Statistics indicate that in 2022, around 268,500 men in the United States received a prostate cancer diagnosis, with about 34,500 succumbing to the illness. This positions prostate cancer as the second most common cause of death among American males [1]. Adenocarcinoma represents one of the most common variants of prostate cancer. The primary risk factors associated with the onset of this malignancy encompass dietary patterns, obesity, smoking habits, levels of physical activity [2], as well as racial and ethnic backgrounds and geographical distribution [3]. Additionally, a familial history of prostate cancer or related conditions such as hereditary breast and ovarian cancers, Lynch syndrome [4], along with infections caused by HPV and EBV [5], are also correlated with an elevated risk for developing prostate cancer. Recent advancements in tumor detection technology have significantly enhanced the early clinical diagnosis of prostate cancer, resulting in a notable reduction in its mortality rate over the past two decades.

The principal treatment modalities for prostate cancer encompass surgical intervention, cryotherapy, radiation therapy, chemotherapy, hormone therapy, and various combination approaches [6]. Resistance represents a significant hurdle in the efficacy of various cancer therapies, frequently resulting in the development of resistance or hormone insensitivity in cancer cells following an initial favorable response to treatment, ultimately leading to recurrence. Prostate cancer generally exhibits a positive response to hormone therapy; however, it invariably progresses to either resistance or castration-resistant forms [7, 8]. Thereby transforming into CRPC and NEPC (Table 1). Additionally, DNPC (Table 1) is a kind of prostate cancer that is independent of both androgen receptor and neuroendocrine signals. DNPC is more invasive and more prone to metastasis, such as DU 145, PC-3, etc. Current therapeutic strategies are inadequate for effectively managing the progression of advanced and metastatic prostate cancer. In recent years, as our comprehension of molecular biology has deepened, gene therapy has emerged as a pivotal approach across various medical disciplines, indicating its potential as a promising direction for future treatment modalities.

**Table 1:** This table presents the full forms of some abbreviations that have appeared in this text

Abbreviation	Full name
HMG	High mobility group
ESC	Embryonic stem cell
IHH	Idiopathic hypogonadotropic hypogonadism
EMT	Epithelial–Mesenchymal transition
CIgG	Cancer-derived Immunoglobulin G
ADT	Androgen deprivation therapy
CRPC	Castration-resistant prostate cancer
LSD1	Lysine specific demethylase 1
MR	Main regulatory factor
ADPC	Adenocarcinoma prostate cancer
NEPC	Neuroendocrine prostate cancer
HRPC	Hormone-refractory prostate cancer
CLC-3	Chloride ion voltage-gated channel 3
SOCE	Store-operated calcium entry
ER	Endoplasmic reticulum
CSC	Cancer stem cell
AR	Androgen receptor
TME	Tumor microenvironment
DNPC	Double-negative prostate cancer

A variety of genes are associated with the development of prostate cancer, including SOX2, ETS, MYC, TP53, RB1, PTEN and SPOP [9]. Among these, SOX2 stands out as the most prominent member of the SOX gene family and is located on chromosome 3 at regions q26.3–27. This gene encodes a protein comprising 317 amino acids [10]. A substantial body of research demonstrates a significant correlation between SOX2 and prostate cancer. SOX2 enhances the progression, invasion, and metastasis of prostate cancer through various mechanisms, while also playing a role in the development of drug resistance. SOX2 not only acts as a crucial biomarker for lymph node metastasis in prostate cancer but also serves as an important therapeutic target for this condition [11].

This manuscript will analyze the structure and physiological functions of SOX2 in the context of prostate cancer, while concurrently investigating the associated signaling pathways. This inquiry seeks to deepen our understanding of SOX2's role in prostate cancer and to identify more effective therapeutic strategies.

## The structural characteristics of SOX2

**Fig. 1:**
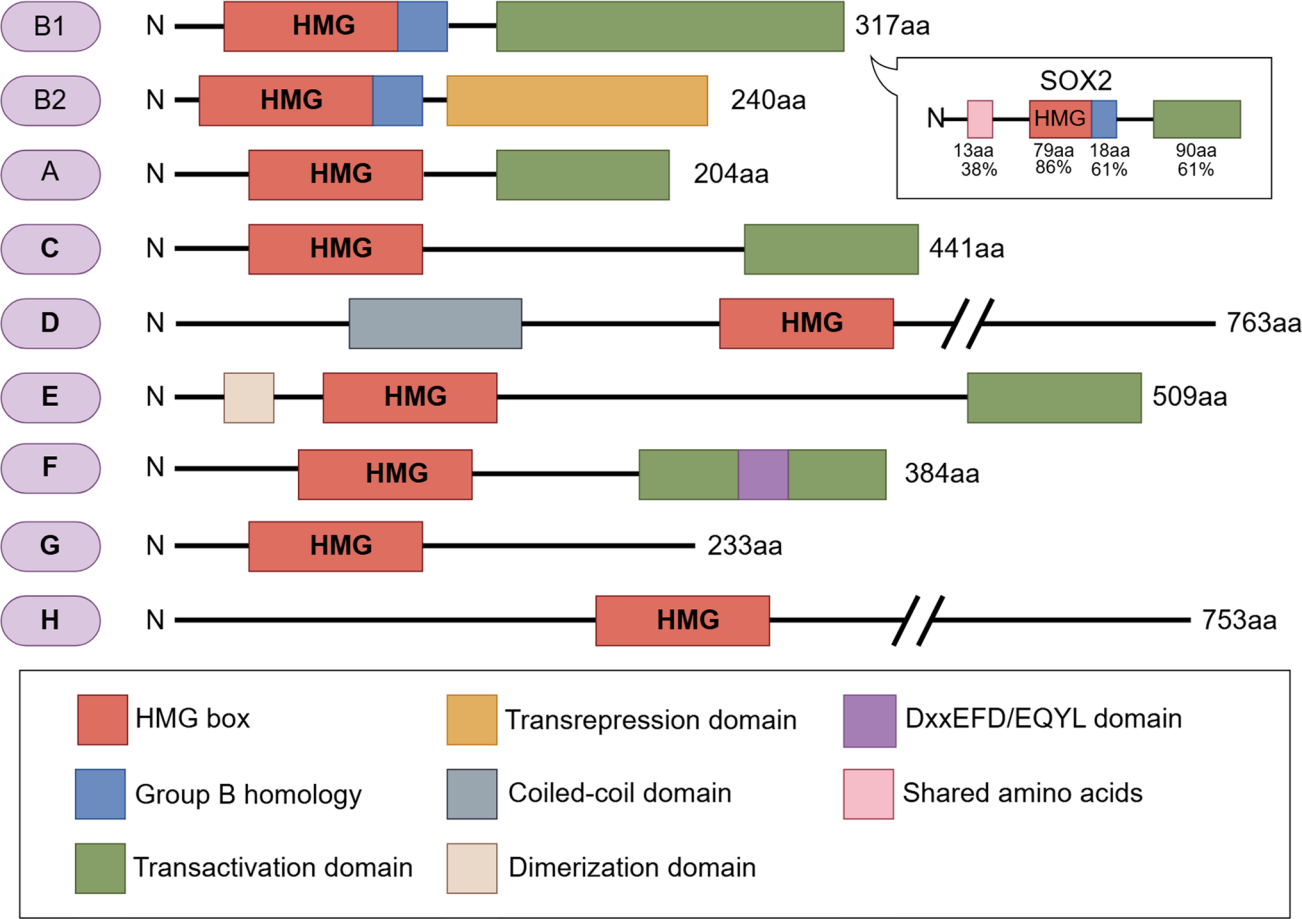
Structural diagram of SOX2. The SOX protein family is categorized into eight distinct classes, designated from A to H, with SOX2 classified as a member of the SOXB1 subfamily. This illustration was generated utilizing Figdraw

The SOX transcription factor is a DNA-binding protein characterized by the presence of an HMG (Table 1) box, which is linked to the SRY (sex-determining region Y) [12]. The SOX protein associates with the TTGTT DNA sequence through three alpha helices within its HMG domain, forming an L-shaped structural motif that interacts with the minor grooves of the DNA [13]. The variability of the HMG domain outside the amino acid sequence of SOX proteins facilitates their interaction with diverse transcription factors and regulatory elements, thereby modulating a spectrum of cellular functions and developmental processes. The SOX protein family comprises over 20 members, which are classified into eight categories (A to H) based on structural and sequence homology within the HMG box [14]. Each category encompasses 17–20 sub-types, with members exhibiting not only overlapping functions but also analogous biochemical characteristics. SOX2 is a member of the SOXB1 subfamily, and its gene is located on chromosome 3q26.3-q27. The N-terminal domain of SOX2 contains an HMG box, while its C-terminal domain associates with other transcription factors (Fig. 1).

SOX1, SOX2, and SOX3 are constituents of the SOXB1 subfamily, demonstrating considerable sequence similarity both within and beyond their HMG box. The architecture of the SOXB1 proteins encompasses a HMG domain that facilitates DNA binding, which serves as a hallmark feature of the SRY related HMG box transcription factor family. This domain empowers the SOXB1 proteins to bind to specific DNA sequences and regulate the expression of target genes. Moreover, members of the SOXB1 subfamily contain a B-like homology domain positioned adjacent to the HMG box, in addition to a trans-activation domain at their C terminus [11] (Fig. 1). These characteristics are integral to their transcription activity and facilitate interactions with other proteins or regulatory elements within the cellular environment.

## Function of SOX2 in physiological mechanisms

SOX2 is expressed during the early phases of embryonic development, and its absence may result in embryonic mortality [10]. SOX2 is primarily localized in the nuclei of ESC (Table 1) and is recognized as one of the earliest markers for the inner cell mass in mammalian embryos. SOX2 is essential for the maintenance of pluripotency and self-renewal in ESC [15].

Research demonstrates that SOX2 is essential for maintaining the stem cell properties and self-renewal potential of embryonic cells, as well as various adult stem cell populations. Furthermore, SOX2 exerts a specific regulatory influence during the early stages of differentiation in embryonic stem cells [16]. SOX2 is integral to maintaining the characteristics of ESC and promoting their differentiation into trophoblast cells. The maintenance of pluripotency in stem cells is regulated by a core transcription network comprising SOX2, OCT4, and Nanog. They typically act as activators of genes encoding cofactors, transcription factors, and chromatin regulators. Among them, both SOX2 and OCT4 transcription factors fall within the Yamanaka factor family. The Yamanaka factors are conspicuously expressed in ESC and can restore the pluripotency of differentiated cells. In the cancer context, these factors have the capacity to reprogram cancer cells into a pluripotent state, contributing to the maintenance of the diversity of tumor cells [17]. These three factors collaboratively enhance the expression of pluripotent genes (including Nanog, OCT4, and SOX2) while concurrently repressing genes associated with differentiation [10]. SOX2 is essential for the development of cell lineages related to the endoderm, ectoderm and mesoderm [18]. Research suggests that SOX2 can regulate the expression of downstream target genes through specific interactions with the HMG gene via its HMG binding domain [19]. This interaction is vital for maintaining stem cell pluripotency. SOX2 is essential for maintaining developmental potential, and its expression levels significantly impact the proliferation of fetal cells [20].

SOX2 is critical for embryonic development in various regions of the nervous system. During later developmental stages, SOX2 is expressed not only in specific neurons and glial cells, but also throughout neural epithelial cells (undifferentiated progenitor stem cells) that play a role in forming the developing neural tube. Consequently, the expression of SOX2 indicates the onset of nervous system formation from the neural induction phase [21].

SOX2 serves as a vital regulator in the development of acinar cells within salivary glands. Research indicates that SOX2 specifically influences genes linked to acinar cell formation, and its absence results in a marked decrease in the production of these cells. The function of SOX2 in this context is influenced by the parasympathetic nervous system and is maintained through the acetylcholine-muscarinic-calcium signaling pathway [22].

Additionally, SOX2 is expressed in the olfactory epithelial cells of adults, primarily localized to sustentacular cells, horizontal basal cells, and specific spherical basal cells. In the context of olfactory basal cell proliferation and their differentiation into neurons, SOX2 plays a pivotal regulatory role [23].

## The role of SOX2 in pathology

The transcription factor SOX2 is integral to embryonic development, and its dysfunction can lead to a variety of functional disorders or the onset of cancer. In humans, mutations in the SOX2 gene are linked to multiple central nervous system abnormalities, including visual impairment, Hippocampus injury, intellectual disabilities, and motor control deficits [21]. Invest igations have revealed that a nonsense mutation in the SOX2 gene can result in IHH [24] (Table 1). This disorder is characterized by manifestations such as anophthalmia, microphthalmia, or coloboma, along with a range of neurological complications including epilepsy [25]. Furthermore, the up-regulation of SOX2 and gene amplification are closely linked to the initiation and progression of various cancer types. Evidence suggests that the transcription factor SOX2 exerts a positive effect on cancer cell activities, including proliferation, invasion, migration, EMT (Table 1), as well as spheroid and colony formation. In cancer cells, SOX2 is integral to the regulation of stem cell characteristics and cellular proliferation, which are regarded as critical factors driving tumor occurrence. SOX2 is significantly over-expressed in CSC [26] (Table 1). Studies have demonstrated that while CSC lack androgen receptor expression, they express embryonic markers such as SOX2, NANOG, OCT4 and the surface marker CD44. Moreover, CSC exhibit the capacity for self-renewal and can generate new tumor cells. SOX2 contributes to the up-regulation of CSC expression and promotes cancer cell invasion through mechanisms including EMT, anti-apoptotic pathways or pro-survival signaling cascades, ATP-binding cassette-like drug transporters, lineage plasticity, and evasion of immune surveillance.

Furthermore, aberrant expression levels of SOX2 are associated with heightened invasive and metastatic capabilities in these tumor cells. By modulating the cell cycle, anti-apoptotic pathways, and cell adhesion mechanisms, SOX2 may enhance the adaptability of tumor cells to their surrounding environment. SOX2 is acknowledged as a tumor promoter across various cancer types, including prostate [27] and cervical cancers [28], primarily by maintaining the stem-like properties of cancer cells that drive tumor progression and dissemination. In contrast, in certain specific malignancies such as gastric cancer [29], SOX2 may instead function as a tumor suppressor. Research indicates that the levels of SOX2 dosage are significantly associated with both the advancement of tumor cells and their dormant state [20]. Ethan P Metz and colleagues have elucidated that the role of SOX2 in various tumor cell types, including prostate cancer, adenocarcinoma of the Pancreatic Duct, and medulloblastoma cells, is concentration-dependent [30]. Specifically, when SOX2 levels attain physiological ranges, it promotes tumor proliferation and metastasis; conversely, exceeding these endogenous levels in vivo results in the suppression of tumor growth and spread. These findings prompt further exploration of innovative strategies for targeting dormant cancer cells.

## The expression of SOX2 in prostate cancer tissues

Research has demonstrated that the levels of SOX2 expression in prostate cancer tissues are markedly higher than those found in normal prostate tissues, and this elevated expression correlates with the prognosis of prostate cancer [31–33] (Table 2). SOX2 is predominantly localized in basal epithelial cells of normal and benign prostate tissues. Conversely, its expression undergoes alterations in prostate cancer, demonstrating a more uniform distribution. This shift in expression pattern may facilitate the classification of tumors into distinct sub-types [34]. SOX2 mRNA and protein are found in most prostate cancer cells. However, the expression of SOX2 shows significant variability within this type of cancer, with markedly higher levels of SOX2 mRNA and protein identified in androgen-independent cell lines such as DU 145, PC-3, PC-3 M, PC-3 M-1 E8, PC-3 M-2B4, NCI-H660, 42D-ENZR, C4-2 ENZR, LNCaP abl, LASCPC and MSK-EF1 [35–37]. In contrast, LNCaP is a cell line that is androgen-dependent exhibits lower levels of SOX2 expression [34, 35]. The observed disparity may indicate the unique biological traits associated with androgen-independent compared to androgen-dependent prostate cancer. The increased levels of SOX2 in androgen-independent prostate cancer are likely linked to its functions in maintaining stem cell-like properties, promoting cellular growth, and influencing cell migration. SOX2 is found not only in most castration-resistant metastatic prostate lesions but also within castration-sensitive prostate cancer cells. Its expression alone can drive the emergence of castration-resistant tumors [34].

**Table 2:** Relationship between SOX2 expression and clinical parameters in prostate cancer

Specimen	Sample size	Methods	Gleason	Survival rate	Age	Grade	Other outcomes	Correlation degree	References
Tissue	80 (40/40)	IHC/WB	+	unk	unk	unk	Hypoxia	+	[36]
Tissue	33 (15/18)	IHC/qRT-PCR	unk	+	unk	+	unk	unk	[29]
Tissue	45/16	IHC/WB	−	unk	unk	unk	Notch1	+	[37]
Tissue	100	IHC	unk	+	unk	+	PD-L1	+	[31]
Tissue	224	IHC/WB	+	unk	unk	+	unk	unk	[85]
Tissue	142	IHC	unk	unk	unk	−	Lymph node involvement	+	[24]
Tissue	87 (16/20/51)	IHC	+	unk	+	+	unk	unk	[86]
Tissue	24/19	IHC/WB	unk	unk	unk	unk	unk	+	[60]
Cell		WB	unk	unk	unk	unk	Metastases	+	[39]

[unk] = unknown, [ +] = positive efect, [−] = no efect

## The function of SOX2 in prostate cancer

SOX2 correlates with prostate cancer progression, invasion and metastasis [27]. It is essential for the stem cell-like traits of prostate cancer, where its elevated expression is associated with sustaining self-renewal and differentiation abilities in tumor stem cells. Furthermore, SOX2 is intricately linked to the metastatic capabilities of prostate cancer cells; it may affect their migration and invasion behaviors while enhancing the adaptability of tumor cells to external conditions, thus promoting metastasis and invasion. Research suggests that reduced levels of SOX2 can impede the growth and invasion of cancer cells while promoting their re-differentiation [38]. Furthermore, it has been shown that SOX2 contributes to castration resistance and lineage plasticity in prostate cancer.

The research indicated that SOX2 expression levels were notably elevated in prostate cancer tissues when compared to those with Inflammation of the prostate gland and benign prostatic hyperplasia. Analyzing the SOX2 expression across various Gleason grades of prostate cancer revealed a statistically significant increase in the proportion of positive SOX2 cells as the grade progressed from 3 to 5. Given that higher Gleason grades correlate with worse prognosis, it is suggested that SOX2 may play a role in the development of prostate cancer and could be significant in its clinical progression [39] (Table 2).

## The TME related to SOX2 in PC

During the course of tumor progression, TME (Table 1) presents remarkable heterogeneity, and the inflammatory TME lays down favorable conditions for various pro-tumor processes, such as vascular generation and EMT. Research findings have suggested that the factors secreted by M1 macrophages with pro-inflammatory features not only raise the level of SOX2 through the NFκB signal transduction pathway, but also down-regulate the AR signaling pathway in prostate cancer cells, thereby giving rise to the plasticity of prostate cancer stem cells [40]. Prostate cancer cells react to the signals emitted by the TME, mainly TGF-β2, BMP-7, GAS6 and Wnt-5a, which can be conveyed via SOX2 and other transcription factors related to pluripotent stem cells. These signals might also influence the Epigenetics by means of histone modifications, thereby triggering dormancy in prostate cancer cells [41]. TME also induces the augmented glycolytic metabolism of prostate cancer stem/progenitor cells and gives rise to acidosis, thereby inducing the tumors to be in a hypoxic state and prompting tumor fibroblasts to release a variety of soluble factors that facilitate tumor growth, angiogenesis, and metastasis [42]. Furthermore, these molecular conversion events might also result in an increase in the expression levels of stem-like gene products like SOX2, Oct3/4 and Nanog in prostate cancer cells [43].

## The mechanism of SOX2 inducing prostate cancer

**Fig. 2:**
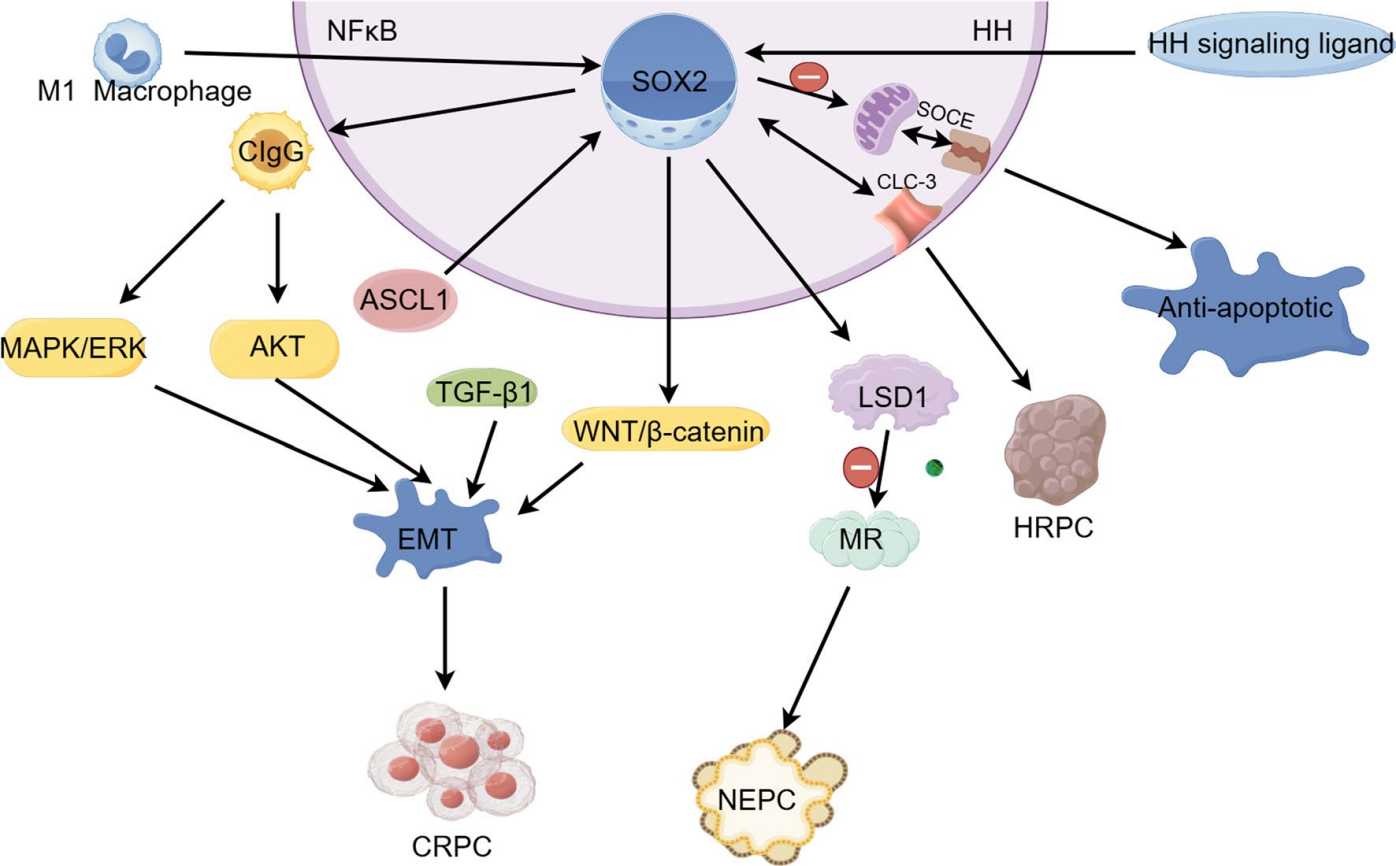
The signaling pathways associated with SOX2 in the context of prostate cancer. SOX2 can participate in the lineage conversion of prostate cancer through multiple signaling pathways and increase the anti-apoptotic characteristics of prostate cancer. This illustration was generated utilizing Figdraw

SOX2 is considered to enhance the proliferation and survival of prostate cancer cells. It may aid in tumor growth and spread by modulating the expression of key genes and impacting cell cycle progression. Below are several ways in which SOX2 plays a role in the advancement of prostate cancer. The relevant mechanisms through which SOX2 contributes to the progression of prostate cancer are summarized in Fig. 2.

### MAPK/ERK and AKT

The study indicates that, besides IgG generated by B cells, cancer cells can also produce CIgG (Table 1) [44]. CIgG is crucial for cancer cells, as it not only improves their attachment to the extracellular matrix but also promotes adhesion between neighboring cancer cells. ADT (Table 1) induces the expression of CIgG in prostate cancer, with SOX2 playing a crucial role in its up-regulation. This enhancement promotes the progression of prostate cancer by activating the MAPK/ERK and AKT signaling pathways, which facilitate EMT and lineage plasticity [45] (Fig. 2). Ultimately, this mechanism contributes to the advancement of prostate cancer into CRPC (Table 1) [46, 47].

### WNT/β-catenin

The elevated expression of SOX2 in prostate cancer cells promotes the EMT, with TGF-β1 further amplifying this phenomenon. EMT is defined by a reduction in cell adhesion properties among epithelial cells, along with an increase in motility [48], which enhances the metastatic capabilities of prostate cancer. Research has shown that in prostate cancer, SOX2 can suppress E-cadherin expression by activating the WNT/β-catenin signaling pathway, which facilitates EMT [49, 50]. Additionally, it has been established that SOX2 interacts with the promoter region of β-catenin, indicating its direct role in regulating this gene's transcription and contributing to the metastasis of prostate cancer (Fig. 2).

### LSD1-MR

SOX2 has the ability to facilitate the transformation of prostate cancer lineages. In this process, SOX2 functions as a potential inhibitor by enhancing the expression of LSD1 (Table 1) [51]. This action leads to significant global lower methylation of histone H3 [52], which in turn suppresses MR (Table 1) associated with ADPC (Fig. 2). Consequently, this results in the transition of prostate cancer from ADPC (Table 1) to aggressive NEPC (Table 1) [53]. The study revealed that ASCL1, a transcription factor oriented towards neuronal lineage, serves as a key driver for the transcription of plastic neurons within this lineage and directly modulates SOX2, promoting the differentiation of prostate cancer into NEPC. Additionally, the expression of SOX2 is reduced when ASCL1 is knocked down [54].

### HH-SOX2

Recent studies have demonstrated that in prostate cancer, both SOX2 and components of the Hedgehog signaling pathway (HH) are subject to amplification. The concurrent expression of these two factors is significantly associated with the aggressiveness seen in advanced stages of prostate cancer. Furthermore, it has been confirmed that SOX2 functions as a downstream target gene within the HH signaling cascade [31] (Fig. 2). As a result, targeting the HH-SOX2 axis therapeutically has shown more favorable outcomes compared to mono-therapy approaches, including reduced cell proliferation, complete inhibition of migration, and increased chromatin condensation [31]. Although this strategy necessitates further validation through in vivo experiments, co-targeting transcription factors along with Carcinogenic signaling pathways presents a promising new avenue for prostate cancer treatment.

### SOX2/CLC-3

CLC-3 (Table 1) is integral to the regulation of the cell cycle in prostate cancer cells [55]. Studies demonstrate that SOX2 can co-immunoprecipitate with CLC-3, and conversely, CLC-3 can also co-immunoprecipitate with SOX2, indicating a reciprocal regulatory interaction between these two proteins (Fig. 2). Therefore, it is clear that SOX2 engages with CLC-3 to collaboratively modulate the cell cycle. Decreased expression levels of CLC-3 and SOX2 result in the arrest of the DU145 cell line, which is representative of HRPC (Table 1), in the G0/G1 phase. Thus, SOX2 can interact with CLC-3 and regulate the cell cycle, which is essential for the initiation and maintenance of tumorigenesis in prostate cancer [56].

### SOX2-SOCE

SOCE (Table 1), characterized by calcium-activated Ca^2+^ influx, is essential for the regulation of various physiological and pathological processes, including apoptosis, through the management of intracellular and extracellular calcium concentrations. SOCE is mediated by specialized channels known as SOC, with Orai1 and STIM1 serving as key components. Orai1 is situated in the plasma membrane functioning as a gatekeeper, while STIM1 resides in the ER (Table 1) membrane where it senses diminished ER calcium levels. The interaction between STIM1 and Orai1 subsequently initiates SOCE activation [57–59]. Research suggests that a reduction in SOCE function may enhance apoptosis resistance in prostate cancer cells. Conversely, SOX2 can augment the anti-apoptotic characteristics of these cells by down-regulating SOCE activity [60] (Fig. 2).

### SOX2 is involved in metabolic pathways

SOX2 is capable of enhancing various metabolic pathways by increasing mitochondrial numbers [61], which subsequently elevates glycolytic capacity and lactate levels. Notably, this effect is observed solely during the metastatic stage of prostate cancer [62], indicating a potential association with prostate cancer metastasis.

## SOX2 is associated with other types of tumors

SOX2 is integral to the advancement of prostate cancer, and its functional disorder has been linked to a variety of other malignancies. Furthermore, SOX2 plays a significant role in enhancing the proliferation, migration, invasion, and metastasis of cancer cells [10]. SOX2 has been shown to enhance the growth and spread of cancer cells in various types of tumors, including prostate cancer [11], lung cancer [63], laryngeal cancer [64], cervical cancer [7], liver cancer [8], and breast cancer [65]. Conversely, in some other cancers, such as gastric cancer [29], it appears to suppress the proliferation and dissemination of tumor cells. Generally, the expression of SOX2 is diminished in gastric cancer [29]. Research suggests that elevated levels of SOX2 can inhibit distant metastasis in gastric cancer primarily by reducing lymph node involvement. Furthermore, there exists a significant association between SOX2 expression levels and poor survival outcomes along with unfavorable prognoses for patients with gastric cancer; consequently, those with more favorable outcomes tend to display higher levels of SOX2 expression [66].

In cervical cancer, SOX2 plays a crucial role in promoting the formation of tumor spheres [28], and its expression is significantly correlated with the prognosis of cervical squamous cell carcinoma [7]. SOX2 is not only implicated in the initiation, progression, invasion, and metastasis of breast cancer but also plays a significant role in its recurrence and resistance [65]. Additionally, studies have demonstrated that SOX2 contributes to the progression of laryngeal cancer, where it exerts crucial regulatory effects on apoptosis and metastasis of laryngeal cancer cells through the MAP4K4/JNK signaling pathway [64]. SOX2 is not only frequently over-expressed across various cancer types, but it also modulates the physiological functions of cancer cells through complex protein–protein interactions and cellular signaling pathways [67].

The expression of SOX2 is positively associated with the aggressive of hepatocellular carcinoma and functions as an independent prognostic marker for this malignancy [8]. Studies have demonstrated that SOX2 not only facilitates the EMT in hepatocellular carcinoma cells, but is also correlated with poor survival outcomes and metastasis in patients diagnosed with hepatocellular carcinoma [68]. SOX2 plays a critical role in the initiation and progression of lung cancer. Research has demonstrated that SOX2 similarly modulates the expression levels of WNT1, WNT4, NOTCH2, and c-MYC in this malignancy [69]. Importantly, while SOX2 promotes tumor progression in lung cancer, it also functions as a significant biomarker linked to favorable patient outcomes [63]. Furthermore, increased expression of SOX2 may serve as a prognostic indicator for individuals undergoing surgical intervention at stages I and II of lung cancer [70]. Thus, SOX2 presents both an adverse role in facilitating tumor growth and a positive aspect concerning treatment efficacy.

## The role of SOX2 in prostate cancer therapy

A variety of mechanisms are implicated in the onset of CRPC, with a predominant focus on the AR (Table 1). As a result, considerable attention in prostate cancer research has been directed toward inhibiting AR signaling to mitigate the development of CRPC. Recent technological advancements have led to significant progress in targeted gene therapy, and our comprehension of SOX2's role in prostate cancer has similarly deepened. Thus, exploring SOX2 as a viable therapeutic target is of paramount importance.

### Lineage plasticity and treatment resistance

The up-regulation of SOX2 in prostate cancer is associated with lineage plasticity, defined as the tumor's ability to transition between distinct developmental pathways. This phenomenon underlies internal heterogeneity and equips tumors to adapt to adverse environments, thereby facilitating their evolution into various histology sub-types while diminishing reliance on the original carcinogenic drivers, ultimately leading to treatment resistance [71]. Prostate cancer cells can develop resistance to androgen-targeting therapies through a phenotypic transition from AR-dependent epithelial cells to AR-independent basal-like cells [72]. Research indicates that the elevated expression level of SOX2 is capable of driving the functional loss of TP53 and RB1, thereby facilitating lineage plasticity. In reality, lineage plasticity is driven by multiple transcription factors and epigenetic regulatory factors. Besides the mutations or deletions of TP53 and RB1, it is also characterized by the loss of AR and AR target genes and the over-expression of MYCN (encoding N-MYC on chromosome 2p24) and AURKA (encoding Aurora-A) [73–75], along with the reduction of PKC-lambda/iota [76], the increased expression of HP1α [77], the over-expression of SRRM4 [78], and the hypoxia-mediated signaling pathway [79, 80].

### SOX2-CIGG and treatment resistance

The progression of prostate cancer is predominantly driven by androgen, which has resulted in the widespread application of ADT as a treatment modality. Nevertheless, ADT demonstrates efficacy primarily in the early stages of prostate cancer, while its effectiveness diminishes significantly in advanced phases. This reduced efficacy is attributed to an increase in SOX2 expression following ADT, which subsequently activates the CIGG pathway that can promote further tumor development. As a result, heightened levels of SOX2 during late-stage ADT are associated with the emergence of drug resistance [26]. Moreover, SOX2 is capable of disrupting the cell cycle, which results in resistance to nuclear hormone receptor inhibitors in prostate cancer. Therefore, down-regulating SOX2 expression in prostate cancer cell lines can markedly improve their sensitivity to androgen receptor signaling inhibitors [81].

### SOX2-PI3K/Akt and treatment resistance

SOX2 has been demonstrated to enhance the resistance of prostate cancer cells to paclitaxel through the PI3K/Akt signaling pathway, promote cellular proliferation, and display anti-apoptotic characteristics, thereby facilitating the emergence of hormone-refractory prostate cancer [82]. Moreover, in light of SOX2's role, we suggest that its expression may serve as a crucial biomarker for predicting therapeutic responses to paclitaxel [83].

## Discussion

SOX2 plays a pivotal role in the molecular mechanisms associated with prostate cancer. The Wnt/β-catenin pathway, along with miR-369-3p/CFL2 and SOX2-CIgG interactions, presents promising therapeutic targets for this malignancy. Furthermore, CIgG and TGF-α may serve as viable treatment options. To reduce the proliferation and survival of prostate cancer cells, we can design specific small molecule inhibitors that target SOX2 activity, effectively suppressing its expression or function. Additionally, we can employ small interfering RNA (siRNA) or short hairpin RNA (shRNA) techniques to specifically silence the SOX2 gene, leading to a decrease in its expression and subsequently inhibiting the growth of prostate cancer.

## Conclusion

This paper presents a comprehensive examination of the structure and function of SOX2, its involvement in signaling pathways related to prostate cancer, and its implications for treatment strategies. The primary objective is to provide a theoretical framework for enhancing therapeutic approaches to prostate cancer. As previously discussed, it is clear that SOX2, as an essential member of the SOX family, not only plays a significant role in embryonic development but also exerts complex effects on the initiation, progression, proliferation, and metastasis of prostate cancer as well as other specific malignancies. As previously discussed, SOX2 promotes EMT and lineage plasticity through the activation of the MAPK/ERK and AKT signaling pathways. Furthermore, SOX2 enhances EMT by down-regulating E-cadherin expression via the Wnt/β-catenin pathway, thereby facilitating the migration of prostate cancer cells. It also drives lineage transformation by increasing LSD1 expression, which results in a global lower methylation of histone H3. In addition, SOX2 interacts with CLC-3 to modulate cell cycle dynamics, thus initiating and sustaining tumorigenesis in prostate cancer. Moreover, it augments the anti-apoptotic characteristics of prostate cancer cells by diminishing SOCE activity while inducing metabolic pathway alterations that promote metastasis. Furthermore, the expression levels of SOX2 have been shown to be inversely associated with chemotherapy resistance and poor prognostic outcomes in prostate cancer [84]. Therefore, a more thorough understanding of the role of SOX2 in tumors may facilitate the elucidation of molecular regulatory networks involved in tumorigenesis and progression, thereby providing a vital theoretical framework for developing more effective targeted therapeutic strategies.

Over the past decade, substantial progress has been made in elucidating the function of SOX2. However, further exploration of its signaling pathways in relation to prostate cancer is essential. Ongoing clinical trials are currently evaluating targeted SOX2 therapy for prostate cancer, and additional clinical data will be required in the future to validate its efficacy and safety. Targeting SOX2 offers significant potential for developing effective therapies not only for prostate cancer, but also for other tumors, thereby facilitating innovative approaches to cancer treatment.

## Data Availability

No datasets were generated or analysed during the current study.

## References

[1] Pinsky PF, Parnes H. Screening for prostate cancer. N Engl J Med. 2023;388(15):1405–14. 10.1056/NEJMcp2209151. 37043655 10.1056/NEJMcp2209151

[2] Matsushita M, Fujita K, Nonomura N. Influence of diet and nutrition on prostate cancer. Int J Mol Sci. 2020. 10.3390/ijms21041447. 32093338 10.3390/ijms21041447 PMC7073095

[3] Rebbeck TR. Prostate cancer genetics: variation by race, ethnicity, and geography. Semin Radiat Oncol. 2017;27(1):3–10. 10.1016/j.semradonc.2016.08.002. 27986209 10.1016/j.semradonc.2016.08.002 PMC5175208

[4] Vietri MT, et al. Hereditary prostate cancer: genes related, target therapy and prevention. Int J Mol Sci. 2021. 10.3390/ijms22073753. 33916521 10.3390/ijms22073753 PMC8038462

[5] Abidi SH, et al. Viral etiology of prostate cancer: genetic alterations and immune response. A literature review. Int J Surg. 2018;52:136–40. 10.1016/j.ijsu.2018.02.050. 29496646 10.1016/j.ijsu.2018.02.050

[6] Sekhoacha M, et al. Prostate cancer review: genetics, diagnosis, treatment options, and alternative approaches. Molecules. 2022. 10.3390/molecules27175730. 36080493 10.3390/molecules27175730 PMC9457814

[7] Hou T, et al. Putative stem cell markers in cervical squamous cell carcinoma are correlated with poor clinical outcome. BMC Cancer. 2015;15:785. 10.1186/s12885-015-1826-4. 26499463 10.1186/s12885-015-1826-4 PMC4619529

[8] Zheng YW, Nie YZ, Taniguchi H. Cellular reprogramming and hepatocellular carcinoma development. World J Gastroenterol. 2013;19(47):8850–60. 10.3748/wjg.v19.i47.8850. 24379607 10.3748/wjg.v19.i47.8850 PMC3870535

[9] Yu C, et al. From genomics to functions: preclinical mouse models for understanding oncogenic pathways in prostate cancer. Am J Cancer Res. 2019;9(10):2079–102. 31720076 PMC6834478

[10] Novak D, et al. SOX2 in development and cancer biology. Semin Cancer Biol. 2020;67(Pt 1):74–82. 10.1016/j.semcancer.2019.08.007. 31412296 10.1016/j.semcancer.2019.08.007

[11] Grimm D, et al. The role of SOX family members in solid tumours and metastasis. Semin Cancer Biol. 2020;67(Pt 1):122–53. 10.1016/j.semcancer.2019.03.004. 30914279 10.1016/j.semcancer.2019.03.004

[12] Pouremamali F, et al. The role of SOX family in cancer stem cell maintenance: with a focus on SOX2. Pathol Res Pract. 2022;231:153783. 10.1016/j.prp.2022.153783. 35121364 10.1016/j.prp.2022.153783

[13] Williams CAC, Soufi A, Pollard SM. Post-translational modification of SOX family proteins: key biochemical targets in cancer? Semin Cancer Biol. 2020;67(Pt 1):30–8. 10.1016/j.semcancer.2019.09.009. 31539559 10.1016/j.semcancer.2019.09.009 PMC7703692

[14] Ito M. Function and molecular evolution of mammalian Sox15, a singleton in the SoxG group of transcription factors. Int J Biochem Cell Biol. 2010;42(3):449–52. 10.1016/j.biocel.2009.10.023. 19909824 10.1016/j.biocel.2009.10.023

[15] Feng R, Wen J. Overview of the roles of Sox2 in stem cell and development. Biol Chem. 2015;396(8):883–91. 10.1515/hsz-2014-0317. 25781683 10.1515/hsz-2014-0317

[16] Kallas A, et al. SOX2 is regulated differently from NANOG and OCT4 in human embryonic stem cells during early differentiation initiated with sodium butyrate. Stem Cells Int. 2014;2014:298163. 10.1155/2014/298163. 24707296 10.1155/2014/298163 PMC3951062

[17] Fatma H, Siddique HR. Pluripotency inducing Yamanaka factors: role in stemness and chemoresistance of liver cancer. Expert Rev Anticancer Ther. 2021;21(8):853–64. 10.1080/14737140.2021.1915137. 33832395 10.1080/14737140.2021.1915137

[18] Sarkar A, Hochedlinger K. The sox family of transcription factors: versatile regulators of stem and progenitor cell fate. Cell Stem Cell. 2013;12(1):15–30. 10.1016/j.stem.2012.12.007. 23290134 10.1016/j.stem.2012.12.007 PMC3608206

[19] Boiani M, Schöler HR. Regulatory networks in embryo-derived pluripotent stem cells. Nat Rev Mol Cell Biol. 2005;6(11):872–84. 10.1038/nrm1744. 16227977 10.1038/nrm1744

[20] Metz EP, Rizzino A. Sox2 dosage: A critical determinant in the functions of Sox2 in both normal and tumor cells. J Cell Physiol. 2019;234(11):19298–306. 10.1002/jcp.28610. 31344986 10.1002/jcp.28610 PMC6662612

[21] Mercurio S, et al. Deconstructing Sox2 function in brain development and disease. Cells. 2022. 10.3390/cells11101604. 35626641 10.3390/cells11101604 PMC9139651

[22] Emmerson E, et al. SOX2 regulates acinar cell development in the salivary gland. Elife. 2017. 10.7554/eLife.26620. 28623666 10.7554/eLife.26620 PMC5498133

[23] Li Z, et al. Sox2 regulates globose basal cell regeneration in the olfactory epithelium. Int Forum Allergy Rhinol. 2022;12(3):286–92. 10.1002/alr.22890. 34569176 10.1002/alr.22890 PMC8860864

[24] Cassin J, et al. Heterozygous mutations in SOX2 may cause idiopathic hypogonadotropic hypogonadism via dominant-negative mechanisms. JCI Insight. 2023. 10.1172/jci.insight.164324. 36602867 10.1172/jci.insight.164324 PMC9977424

[25] Shima H, et al. SOX2 nonsense mutation in a patient clinically diagnosed with non-syndromic hypogonadotropic hypogonadism. Endocr J. 2017;64(8):813–7. 10.1507/endocrj.EJ17-0078. 28659543 10.1507/endocrj.EJ17-0078

[26] Matsika A, et al. Cancer stem cell markers in prostate cancer: an immunohistochemical study of ALDH1, SOX2 and EZH2. Pathology. 2015;47(7):622–8. 10.1097/pat.0000000000000325. 26517640 10.1097/PAT.0000000000000325

[27] Chaudhary S, et al. Sox2: a regulatory factor in tumorigenesis and metastasis. Curr Protein Pept Sci. 2019;20(6):495–504. 10.2174/1389203720666190325102255. 30907312 10.2174/1389203720666190325102255

[28] Wang L, et al. Enrichment and characterization of cancer stem-like cells from a cervical cancer cell line. Mol Med Rep. 2014;9(6):2117–23. 10.3892/mmr.2014.2063. 24676900 10.3892/mmr.2014.2063 PMC4055449

[29] Wang S, et al. SOX2, a predictor of survival in gastric cancer, inhibits cell proliferation and metastasis by regulating PTEN. Cancer Lett. 2015;358(2):210–9. 10.1016/j.canlet.2014.12.045. 25543086 10.1016/j.canlet.2014.12.045

[30] Metz EP, et al. Tumor quiescence: elevating SOX2 in diverse tumor cell types downregulates a broad spectrum of the cell cycle machinery and inhibits tumor growth. BMC Cancer. 2020;20(1):941. 10.1186/s12885-020-07370-7. 32998722 10.1186/s12885-020-07370-7 PMC7528478

[31] Kar S, et al. SOX2 function and Hedgehog signaling pathway are co-conspirators in promoting androgen independent prostate cancer. Biochim Biophys Acta Mol Basis Dis. 2017;1863(1):253–65. 10.1016/j.bbadis.2016.11.001. 27816521 10.1016/j.bbadis.2016.11.001

[32] Sattler HP et al. Novel amplification unit at chromosome 3q25-q27 in human prostate cancer. Prostate. 2000;45(3):207–15. 10.1002/1097-0045(20001101)45:3<207::AID-PROS2>3.0.CO;2-H. 10.1002/1097-0045(20001101)45:3<207::aid-pros2>3.0.co;2-h 11074522

[33] de la Maza MDF, et al. Immune biomarkers in metastatic castration-resistant prostate cancer. Eur Urol Oncol. 2022;5(6):659–67. 10.1016/j.euo.2022.04.004. 35491356 10.1016/j.euo.2022.04.004 PMC7617991

[34] Kregel S, et al. Sox2 is an androgen receptor-repressed gene that promotes castration-resistant prostate cancer. PLoS ONE. 2013;8(1):e53701. 10.1371/journal.pone.0053701. 23326489 10.1371/journal.pone.0053701 PMC3543364

[35] Lin F, et al. Sox2 targets cyclinE, p27 and survivin to regulate androgen-independent human prostate cancer cell proliferation and apoptosis. Cell Prolif. 2012;45(3):207–16. 10.1111/j.1365-2184.2012.00812.x. 22469032 10.1111/j.1365-2184.2012.00812.x PMC6495851

[36] Pham MT, et al. Identifying phased mutations and complex rearrangements in human prostate cancer cell lines through linked-read whole-genome sequencing. Mol Cancer Res. 2022;20(7):1013–20. 10.1158/1541-7786.Mcr-21-0683. 35452513 10.1158/1541-7786.MCR-21-0683 PMC9262859

[37] Ye C, et al. Enzalutamide-resistant related lncRNA NONHSAT210528 promotes the proliferation and invasion of prostate cancer. Transl Androl Urol. 2022;11(5):643–58. 10.21037/tau-22-99. 35693714 10.21037/tau-22-99 PMC9177268

[38] Yu X, et al. SOX2 expression in the developing, adult, as well as, diseased prostate. Prostate Cancer Prostat Dis. 2014;17(4):301–9. 10.1038/pcan.2014.29. 10.1038/pcan.2014.29 PMC4227931 25091041

[39] Aboushousha T, et al. Comparative expression of RAGE and SOX2 in benign and malignant prostatic lesions. Asian Pac J Cancer Prev. 2019;20(2):615–20. 10.31557/apjcp.2019.20.2.615. 30806068 10.31557/APJCP.2019.20.2.615 PMC6897005

[40] Kainulainen K, et al. Secreted factors from M1 macrophages drive prostate cancer stem cell plasticity by upregulating NANOG, SOX2, and CD44 through NFκB-signaling. Oncoimmunology. 2024;13(1):2393442. 10.1080/2162402x.2024.2393442. 39175947 10.1080/2162402X.2024.2393442 PMC11340773

[41] Cackowski FC, Heath EI. Prostate cancer dormancy and recurrence. Cancer Lett. 2022;524:103–8. 10.1016/j.canlet.2021.09.037. 34624433 10.1016/j.canlet.2021.09.037 PMC8694498

[42] Andreucci E, et al. The acidic tumor microenvironment drives a stem-like phenotype in melanoma cells. J Mol Med (Berl). 2020;98(10):1431–46. 10.1007/s00109-020-01959-y. 32803272 10.1007/s00109-020-01959-y PMC7525286

[43] Mimeault M, Batra SK. Frequent gene products and molecular pathways altered in prostate cancer- and metastasis-initiating cells and their progenies and novel promising multitargeted therapies. Mol Med. 2011;17(9–10):949–64. 10.2119/molmed.2011.00115. 21607288 10.2119/molmed.2011.00115 PMC3188882

[44] Qiu X, et al. Human epithelial cancers secrete immunoglobulin g with unidentified specificity to promote growth and survival of tumor cells. Cancer Res. 2003;63(19):6488–95. 14559841

[45] Kinkade CW, et al. Targeting AKT/mTOR and ERK MAPK signaling inhibits hormone-refractory prostate cancer in a preclinical mouse model. J Clin Invest. 2008;118(9):3051–64. 10.1172/jci34764. 18725989 10.1172/JCI34764 PMC2518074

[46] Qin C, et al. Cancer-driven IgG promotes the development of prostate cancer though the SOX2-CIgG pathway. Prostate. 2020;80(13):1134–44. 10.1002/pros.24042. 32628304 10.1002/pros.24042

[47] Sarker D, et al. Targeting the PI3K/AKT pathway for the treatment of prostate cancer. Clin Cancer Res. 2009;15(15):4799–805. 10.1158/1078-0432.Ccr-08-0125. 19638457 10.1158/1078-0432.CCR-08-0125

[48] Huang Y, Hong W, Wei X. The molecular mechanisms and therapeutic strategies of EMT in tumor progression and metastasis. J Hematol Oncol. 2022;15(1):129. 10.1186/s13045-022-01347-8. 36076302 10.1186/s13045-022-01347-8 PMC9461252

[49] Li X, et al. SOX2 promotes tumor metastasis by stimulating epithelial-to-mesenchymal transition via regulation of WNT/β-catenin signal network. Cancer Lett. 2013;336(2):379–89. 10.1016/j.canlet.2013.03.027. 23545177 10.1016/j.canlet.2013.03.027

[50] Savagner P. The epithelial-mesenchymal transition (EMT) phenomenon. Ann Oncol. 2010;21(Suppl 7):vii89-92. 10.1093/annonc/mdq292. 20943648 10.1093/annonc/mdq292 PMC3379967

[51] Zhang X, et al. Pluripotent stem cell protein Sox2 confers sensitivity to LSD1 inhibition in cancer cells. Cell Rep. 2013;5(2):445–57. 10.1016/j.celrep.2013.09.018. 24139802 10.1016/j.celrep.2013.09.018 PMC11162149

[52] Yamada Y, Beltran H. Clinical and biological features of neuroendocrine prostate cancer. Curr Oncol Rep. 2021;23(2):15. 10.1007/s11912-020-01003-9. 33433737 10.1007/s11912-020-01003-9 PMC7990389

[53] Li H, et al. SOX2 has dual functions as a regulator in the progression of neuroendocrine prostate cancer. Lab Invest. 2020;100(4):570–82. 10.1038/s41374-019-0343-5. 31772313 10.1038/s41374-019-0343-5

[54] Nouruzi S, et al. ASCL1 activates neuronal stem cell-like lineage programming through remodeling of the chromatin landscape in prostate cancer. Nat Commun. 2022;13(1):2282. 10.1038/s41467-022-29963-5. 35477723 10.1038/s41467-022-29963-5 PMC9046280

[55] Huang W, et al. Functional expression of chloride channels and their roles in the cell cycle and cell proliferation in highly differentiated nasopharyngeal carcinoma cells. Physiol Rep. 2014. 10.14814/phy2.12137. 25214521 10.14814/phy2.12137 PMC4270222

[56] Chen J, et al. CLC-3 and SOX2 regulate the cell cycle in DU145 cells. Oncol Lett. 2020;20(6):372. 10.3892/ol.2020.12235. 33154770 10.3892/ol.2020.12235 PMC7608052

[57] Liou J, et al. STIM is a Ca2+ sensor essential for Ca2+-store-depletion-triggered Ca2+ influx. Curr Biol. 2005;15(13):1235–41. 10.1016/j.cub.2005.05.055. 16005298 10.1016/j.cub.2005.05.055 PMC3186072

[58] Roos J, et al. STIM1, an essential and conserved component of store-operated Ca2+ channel function. J Cell Biol. 2005;169(3):435–45. 10.1083/jcb.200502019. 15866891 10.1083/jcb.200502019 PMC2171946

[59] Zhang SL, et al. Genome-wide RNAi screen of Ca(2+) influx identifies genes that regulate Ca(2+) release-activated Ca(2+) channel activity. Proc Natl Acad Sci USA. 2006;103(24):9357–62. 10.1073/pnas.0603161103. 16751269 10.1073/pnas.0603161103 PMC1482614

[60] Jia X, et al. SOX2 promotes tumorigenesis and increases the anti-apoptotic property of human prostate cancer cell. J Mol Cell Biol. 2011;3(4):230–8. 10.1093/jmcb/mjr002. 21415100 10.1093/jmcb/mjr002

[61] de Wet L, et al. SOX2 mediates metabolic reprogramming of prostate cancer cells. Oncogene. 2022;41(8):1190–202. 10.1038/s41388-021-02157-x. 35067686 10.1038/s41388-021-02157-x PMC8858874

[62] Verma P, et al. Cancer stem cell in prostate cancer progression, metastasis and therapy resistance. Biochim Biophys Acta Rev Cancer. 2023;1878(3):188887. 10.1016/j.bbcan.2023.188887. 36997008 10.1016/j.bbcan.2023.188887

[63] Wilbertz T, et al. SOX2 gene amplification and protein overexpression are associated with better outcome in squamous cell lung cancer. Mod Pathol. 2011;24:944–53. 10.1038/modpathol.2011.49. 21460799 10.1038/modpathol.2011.49

[64] Yang N, et al. Silencing SOX2 expression by RNA interference inhibits proliferation, invasion and metastasis, and induces apoptosis through MAP4K4/JNK signaling pathway in human laryngeal cancer TU212 cells. J Histochem Cytochem. 2015;63(9):721–33. 10.1369/0022155415590829. 26001828 10.1369/0022155415590829 PMC4804729

[65] Feng X, Lu M. Expression of sex-determining region Y-box protein 2 in breast cancer and its clinical significance. Saudi Med J. 2017;38(7):685–90. 10.15537/smj.2017.7.19372. 28674712 10.15537/smj.2017.7.19372 PMC5556274

[66] Carrasco-Garcia E, et al. Paradoxical role of SOX2 in gastric cancer. Am J Cancer Res. 2016;6(4):701–13. 27186426 PMC4859879

[67] Weina K, Utikal JS. SOX2 and cancer: current research and its implications in the clinic. Clin Transl Med. 2014;3:19–19. 25114775 10.1186/2001-1326-3-19 PMC4126816

[68] Sun C, et al. SOX2 expression predicts poor survival of hepatocellular carcinoma patients and it promotes liver cancer cell invasion by activating Slug. Med Oncol (Northwood, London, England). 2013;30:503. 10.1007/s12032-013-0503-1. 10.1007/s12032-013-0503-1 23430442

[69] Chen S, et al. SOX2 gene regulates the transcriptional network of oncogenes and affects tumorigenesis of human lung cancer cells. PLoS ONE. 2012;7(5):e36326. 10.1371/journal.pone.0036326. 22615765 10.1371/journal.pone.0036326 PMC3352903

[70] Toschi L, et al. Increased SOX2 gene copy number is associated with FGFR1 and PIK3CA gene gain in non-small cell lung cancer and predicts improved survival in early stage disease. PLoS ONE. 2014;9(4):e95303. 10.1371/journal.pone.0095303. 24736592 10.1371/journal.pone.0095303 PMC3988173

[71] Quintanal-Villalonga Á, et al. Lineage plasticity in cancer: a shared pathway of therapeutic resistance. Nat Rev Clin Oncol. 2020;17(6):360–71. 10.1038/s41571-020-0340-z. 32152485 10.1038/s41571-020-0340-z PMC7397755

[72] Mu P, et al. SOX2 promotes lineage plasticity and antiandrogen resistance in TP53- and RB1-deficient prostate cancer. Science. 2017;355(6320):84–8. 10.1126/science.aah4307. 28059768 10.1126/science.aah4307 PMC5247742

[73] Cheng L, et al. Anatomic, morphologic and genetic heterogeneity of prostate cancer: implications for clinical practice. Expert Rev Anticancer Ther. 2012;12(11):1371–4. 10.1586/era.12.127. 23249102 10.1586/era.12.127

[74] Beltran H, et al. Molecular characterization of neuroendocrine prostate cancer and identification of new drug targets. Cancer Discov. 2011;1(6):487–95. 10.1158/2159-8290.Cd-11-0130. 22389870 10.1158/2159-8290.CD-11-0130 PMC3290518

[75] Tan HL, et al. Rb loss is characteristic of prostatic small cell neuroendocrine carcinoma. Clin Cancer Res. 2014;20(4):890–903. 10.1158/1078-0432.Ccr-13-1982. 24323898 10.1158/1078-0432.CCR-13-1982 PMC3931005

[76] Reina-Campos M, et al. Increased serine and one-carbon pathway metabolism by PKCλ/ι deficiency promotes neuroendocrine prostate cancer. Cancer Cell. 2019;35(3):385-400.e9. 10.1016/j.ccell.2019.01.018. 30827887 10.1016/j.ccell.2019.01.018 PMC6424636

[77] Ci X, et al. Heterochromatin protein 1α mediates development and aggressiveness of neuroendocrine prostate cancer. Cancer Res. 2018;78(10):2691–704. 10.1158/0008-5472.Can-17-3677. 29487201 10.1158/0008-5472.CAN-17-3677

[78] Li Y, et al. SRRM4 drives neuroendocrine transdifferentiation of prostate adenocarcinoma under androgen receptor pathway inhibition. Eur Urol. 2017;71(1):68–78. 10.1016/j.eururo.2016.04.028. 27180064 10.1016/j.eururo.2016.04.028

[79] Lin TP, et al. REST reduction is essential for hypoxia-induced neuroendocrine differentiation of prostate cancer cells by activating autophagy signaling. Oncotarget. 2016;7(18):26137–51. 10.18632/oncotarget.8433. 27034167 10.18632/oncotarget.8433 PMC5041970

[80] Ge R, et al. Epigenetic modulations and lineage plasticity in advanced prostate cancer. Ann Oncol. 2020;31(4):470–9. 10.1016/j.annonc.2020.02.002. 32139297 10.1016/j.annonc.2020.02.002

[81] Williams A, et al. SOX2 expression in prostate cancer drives resistance to nuclear hormone receptor signaling inhibition through the WEE1/CDK1 signaling axis. Cancer Lett. 2023;565:216209. 10.1016/j.canlet.2023.216209. 37169162 10.1016/j.canlet.2023.216209 PMC13037384

[82] Li D, et al. Sox2 is involved in paclitaxel resistance of the prostate cancer cell line PC-3 via the PI3K/Akt pathway. Mol Med Rep. 2014;10(6):3169–76. 10.3892/mmr.2014.2630. 25310235 10.3892/mmr.2014.2630

[83] Vaddi PK, et al. Elimination of SOX2/OCT4-associated prostate cancer stem cells blocks tumor development and enhances therapeutic response. Cancers (Basel). 2019. 10.3390/cancers11091331. 31500347 10.3390/cancers11091331 PMC6769476

[84] Wuebben EL, Rizzino A. The dark side of SOX2: cancer—a comprehensive overview. Oncotarget. 2017;8(27):44917–43. 10.18632/oncotarget.16570. 28388544 10.18632/oncotarget.16570 PMC5546531

[85] Bae KM, et al. Hypoxia regulates SOX2 expression to promote prostate cancer cell invasion and sphere formation. Am J Cancer Res. 2016;6(5):1078–88. 27294000 PMC4889721

[86] Du Z, et al. Knocking down SOX2 overcomes the resistance of prostate cancer to castration via notch signaling. Mol Biol Rep. 2023;50(11):9007–17. 10.1007/s11033-023-08757-y. 37716921 10.1007/s11033-023-08757-y

